# Apathy and anhedonia in adult and adolescent cannabis users and controls before and during the COVID-19 pandemic lockdown

**DOI:** 10.1093/ijnp/pyab033

**Published:** 2021-06-02

**Authors:** Martine Skumlien, Christelle Langley, Will Lawn, Valerie Voon, Barbara J Sahakian

**Affiliations:** 1 Department of Psychiatry, University of Cambridge, Cambridge, UK; 2 Behavioural and Clinical Neurosciences Institute, Cambridge, UK; 3 Cambridgeshire and Peterborough NHS Trust, Cambridge, UK; 4 Clinical Psychopharmacology Unit, University College London, London, UK

## Abstract

**Background:**

COVID-19 lockdown measures have caused severe disruptions to work and education, and prevented people from engaging in many rewarding activities. Cannabis users may be especially vulnerable, having been previously shown to have higher levels of apathy and anhedonia than non-users.

**Methods:**

In this survey study, we measured apathy and anhedonia before and after lockdown measures were implemented, in *n*=256 adult and *n*=200 adolescent cannabis users, and *n*=170 adult and *n*=172 adolescent controls. Scores on the Apathy Evaluation Scale (AES) and Snaith-Hamilton Pleasure Scale (SHAPS) were investigated with mixed measures Analyses of Covariance, with factors User-Group, Age-Group, and Time, controlling for depression, anxiety, and other drug use.

**Results:**

Adolescent cannabis users had significantly higher SHAPS scores before lockdown, indicative of greater anhedonia, compared to adolescent controls (*p*=.03, η_p_  ^2^=.013). Contrastingly, adult users had significantly lower scores on both the SHAPS (*p*<.001, η_p_  ^2^=.030) and AES (*p*<.001, η_p_  ^2^=.048) after lockdown, compared to adult controls. Scores on both scales increased during lockdown across groups, and this increase was significantly smaller for cannabis users (AES *p*=.001, η_p_^2^=.014; SHAPS *p*=.01, η_p_^2^=.008). Exploratory analyses revealed that dependent cannabis users had significantly higher scores overall (AES *p*<.001, η_p_  ^2^=.037; SHAPS *p*<.001, η_p_  ^2^=.029), and a larger increase in scores (AES *p*=.04, η_p_^2^ .010; SHAPS *p*=.04, η_p_^2^=.010), compared to non-dependent users.

**Conclusions:**

Our results suggest that adolescents and adults have differential associations between cannabis use, and apathy and anhedonia. Within users, dependence may be associated with higher levels of apathy and anhedonia regardless of age, and a greater increase in levels during the COVID-19 lockdown.

In this study, we show differential associations between cannabis use, and apathy and anhedonia, for adults and adolescents. Adolescent cannabis users had higher levels of anhedonia compared to age-matched controls, whereas adult users had lower levels of apathy and anhedonia compared to age-matched controls. Cannabis dependence was associated with higher levels of apathy and anhedonia within both adult and adolescent users. These results indicate that individual differences within cannabis users may be more important than user status per se, in predicting apathy and anhedonia. We also found that levels of apathy and anhedonia had increased since the onset of the COVID-19 lockdown, and that this increase was larger in dependent compared to non-dependent cannabis users. Our results add to the growing body of evidence on the adverse mental health impact of the COVID-19 lockdown, and highlight groups with potentially increased vulnerability.

### 1. Introduction

The COVID-19 pandemic is a global public health crisis. Lockdown measures intended to mitigate the spread of the virus have imposed a significant constraint on our ability to engage in activities we normally find rewarding, and many have reported feeling a loss of motivation and purpose during the pandemic (Minds, 2020). Meanwhile, the use of cannabis appears to have increased (Winstock et al., 2020). Though the association between cannabis and reward processing is unclear, some previous research has linked cannabis use with reduced motivation (Pacheco-Colón et al., 2018a), placing users at potentially increased risk of anhedonic and amotivational responses to COVID-19 lockdown measures.

Cannabis use has been associated with syndromes of reward and motivation, including apathy (Meier and White, 2018; Petrucci et al., 2020) and anhedonia (Dorard et al., 2008; Leventhal et al., 2017; Lopez-Vergara et al., 2019). Apathy refers to a loss of or reduction in motivation, and anhedonia to a loss of interest in or pleasure from previously rewarding activities (Robert et al., 2009; Treadway and Zald, 2011). One recent large-scale study by Petrucci et al. (2020) found that problematic cannabis use correlated with apathy, and a longitudinal study of over 3000 participants by Leventhal et al. (2017) found that anhedonia predicted future cannabis use during adolescence. Adolescents may be predisposed towards harmful effects of cannabis, including apathy and anhedonia, due to the important neuromaturation that takes place during this time (Bossong and Niesink, 2010; Lubman et al., 2015). However, some studies do not find higher levels of apathy or anhedonia in cannabis users (Dumas et al., 2002; Barnwell et al., 2006; Pacheco-Colón et al., 2018b), thus more research is needed to understand this relationship.

Research on the impact of COVID-19 is in its early stages. Some systematic reviews have suggested a high prevalence of anxiety, depression, and general psychological distress, which has increased since the onset of the pandemic (Li and Wang, 2020; Luo et al., 2020; Pierce et al., 2020; Xiong et al., 2020). Social distancing has meant increased isolation and loneliness, which are important risk factors for mental health problems (Vatansever et al., 2021), and negatively impact social cognition (Bland et al., 2020). These measures may be especially hard on adolescents, given the particular importance of peer interaction during this time (Blakemore, 2008; Kilford et al., 2016). Indeed, several studies suggest that adolescents have faced severe psychosocial consequences due to lockdown (Duan et al., 2020; Loades et al., 2020; Minds, 2020), and that vulnerable adolescents are at particular risk (Fegert et al., 2020). The COVID-19 lockdown could also have potentiated the relationship between cannabis use and apathy and anhedonia. Cannabis users experiencing problems related to abuse and dependence may have had particular difficulty coping with the additional stress brought on by the pandemic. Finally, these vulnerability factors could interact and exacerbate problems (Fegert et al., 2020), thus placing adolescent cannabis users at enhanced risk.

In the current study, we compared levels of apathy and anhedonia in adult and adolescent cannabis users and controls, both before and after COVID-19 lockdown measures were implemented in most European countries in mid-March 2020. The study had two aims. The first was to investigate whether cannabis users, and adolescent users in particular, had higher levels of apathy and anhedonia, compared to controls. With respect to our first aim, we proposed the following hypotheses:

1. Cannabis users will have greater levels of apathy and anhedonia compared to controls before and after lockdown.
2. There will be a larger difference between adolescent users and controls, than between adult users and controls.

The second aim of this study was to investigate whether there had been an increase in levels of apathy and anhedonia as a result of the COVID-19 lockdown, and whether this increase had been more pronounced for cannabis users and adolescents. We proposed the following hypotheses:

3. Levels of apathy and anhedonia will have increased since lockdown measures were implemented in March 2020.
4. This increase will be larger for cannabis users compared to controls.
5. This increase will be larger for adolescent users compared to adult users.

### 2. Methods

*2.1 Participants*

This is an online survey study. The survey was advertised through relevant mailing lists, and on Facebook and Instagram, and responses were collected between 4th of June and 4th of August 2020. Inclusion criteria were being between 16 and 30 years of age, fluent or almost fluent in English, and currently and normally residing in the UK, EU, EEA, or Switzerland. Participants were classified as cannabis users if they had used cannabis minimum four days per month in the three-month period before lockdown was initiated in the UK (i.e. the 23rd of March 2020), and controls if they had used less than this. Adolescents were 16-17 years of age, and adults were 18-30 years of age. Eighteen participants were excluded from analyses (15 did not report levels of depression and/or anxiety and 3 were identified as giving unreliable responses) resulting in a final sample of 798 participants. There were 200 adolescent users, 172 adolescent controls, 256 adult users, and 170 adult controls. Ethical approval was obtained from the University of Cambridge Psychology Research Ethics Committee. All participants provided informed electronic consent to participating in the study. Participants were provided with the option to enter a prize draw of three £100 gift certificates.

*2.2 Measures*

Participants completed an online questionnaire including detailed questions about cannabis and other drug use before and during the COVID-19 lockdown, and changes in life circumstances due to COVID-19. Participants were also asked if they were currently experiencing or had ever experienced a number of different psychiatric disorders, and asked to rate the impact of the pandemic on their mental health on a scale from -5 (a lot worse) to 5 (a lot better). Cannabis dependence was assessed with the Severity of Dependence Scale (SDS; Gossop et al., 1995; Martin et al., 2006).

Anhedonia was measured with the Snaith-Hamilton Pleasure Scale (SHAPS; Snaith et al., 1995). The SHAPS consists of 14 items covering a wide range of pleasurable experiences, and is a reliable and valid measure of anhedonia both in adult (Franken et al., 2007) and adolescent (Leventhal et al., 2015) samples. Apathy was measured with the Apathy Evaluation Scale (AES; Marin et al., 1991). The AES is an 18-item self-report questionnaire with cognitive, behavioural, and emotional dimensions, and has been demonstrated as valid and reliable (Raimo et al., 2014; Lueken et al., 2017). Responses on both scales were coded so that higher scores indicated higher levels of apathy or anhedonia, respectively. Participants completed the AES and SHAPS twice – once answering regarding the three-month period before lockdown, and once answering regarding the past two weeks at the time of responding. Finally, four questions were added to flag inconsistent/unreliable responding.

*2.3 Analyses*

Scores on the SHAPS and AES before and after lockdown served as dependent variables in separate mixed measures Analyses of Covariance (ANCOVAs). Time (before and after lockdown) was the within-subjects factor, and User-Group and Age-Group were between-subject factors. The User-Group*Age-Group, User-Group*Time, Age-Group*Time, and User-Group*Age-Group*Time interactions were also included. Significant effects were followed up with post hoc ANCOVAs. Covariates in all models were days per month of alcohol use, days per month of cigarette use, regular illicit drug use (yes/no), self-reported depression (yes/no), and self-reported anxiety (yes/no), as well as all individual two-way covariate interactions with Time. These covariates were included due to their potential interaction with cannabis use (e.g. Patton et al., 2002; Fergusson et al., 2006; Millar et al., 2021), and to separate apathy and anhedonia from general anxiety and depression. Multiple comparisons correction was performed using the Benjamin-Hochberg false discovery rate (FDR) procedure for AES and SHAPS independently for the effects of interest, with a priori $q < .05$. Partial eta squared ($\eta_p^2$) values were used as measures of effect size. Finally, bivariate Pearson correlations were computed between variables of interest. Analyses were performed using IBM SPSS 27 and R 3.6.2 (R Core Team, 2019).

### 3. Results

*3.1 Sample Characteristics*

Sample characteristics are presented in Table 1. The groups were well balanced with respect to age and gender, and 96.7% were residents in the United Kingdom. Adult users more frequently reported taking medication for a psychiatric or neurological condition (23%) compared to the other groups (9-15%).

[TABLE 1 APPROXIMATELY HERE]

Cannabis use variables are presented in Table 2. Adult users reported using cannabis more days per month, on average, compared to adolescent users, and had a higher average SDS score. Using the recommended cut-off score of 3 for adults and 4 for adolescents (Swift et al., 1998; Martin et al., 2006), $n$ = 130 adults (50.8%) and $n$ = 69 adolescents (34.5%) met the criteria for cannabis dependence. Supplemental figure S1 shows the distribution of days per month of cannabis use for users and controls before and after lockdown. Although the majority retained the same user status, $n$ = 25 controls (7.3%) reported using cannabis more than three days per month after lockdown, and $n$ = 65 cannabis users (14.3%) reported using cannabis less than four days per month after lockdown.

Cronbach's alpha values were 0.86 and 0.90 for AES scores before and after lockdown, respectively, and 0.88 and 0.91 for SHAPS scores before and after lockdown, respectively. Bivariate correlations for variables of interest are presented in Supplemental Table S2.

[TABLE 2 APPROXIMATELY HERE]

*3.2 Main Analyses*

Figure 1 shows mean scores and standard errors on the AES and SHAPS scales for the four groups, before and after lockdown. Supplemental figures S3 and S4 show score distributions for the four groups, before and after lockdown. Full results for AES and SHAPS ANCOVAs, including covariates, are displayed in supplemental tables S5 and S6. All p-values below .05 survived the FDR correction.

*AES*

The repeated measures ANCOVA for AES yielded a significant within-subjects main effect of Time ($F_{1,789} = 83.44$, $p < .001$, $\eta_p^2 = .096$), as well as significant interaction effects for Time*User-Group ($F_{1,789} = 11.22$, $p = .001$, $\eta_p^2 = .014$) and Time*Age-Group ($F_{1,789} = 6.19$, $p = .01$, $\eta_p^2 = .008$), suggesting that scores had increased since lockdown across groups, and that this increase was larger for adolescents and controls than for adults and users. The Time*User*Age interaction was not significant ($F_{1,789} = .70$, $p = .40$). There were also significant between-subjects main effects of User-Group ($F_{1,789} = 11.89$, $p = .001$, $\eta_p^2 = .015$) and Age-Group ($F_{1,789} = 11.27$, $p = .001$, $\eta_p^2 = 014$), and a significant User-Group*Age-Group interaction ($F_{1,789} = 4.19$, $p = .04$, $\eta_p^2 = .005$). Four post hoc univariate ANCOVAs with factor User-Group were conducted separately for adults and adolescents for scores before and after lockdown. There were no significant differences between adolescent users and controls ($F_{1,365} = .02$, $p = .90$ before lockdown, $F_{1,365} = 1.89$, $p = .17$ after lockdown), but adult users scored lower than adult controls both before ($F_{1,419} = 4.00$, $p = .046$, $\eta_p^2 = .009$) and after ($F_{1,419} = 21.10$, $p < .001$, $\eta_p^2 = .048$) lockdown.

*SHAPS*

The repeated measures ANCOVA for SHAPS yielded a marginally significant within-subjects main effect of Time ($F_{1,789} = 3.54$, $p = .06$, $\eta_p^2 = .004$), and a significant Time*User-Group interaction effect ($F_{1,789} = 6.49$, $p = .01$, $\eta_p^2 = .008$) in the direction of greater increase in scores from before to after lockdown for controls compared to users. Figure 1 shows that scores increased by only a marginal amount for users. The Time*Age and Time*User*Age interaction effects were not significant ($F_{1,789} = .07$, $p = .80$ and $F_{1,789} = .28$, $p = .60$, respectively). There were also significant between-subjects effects of Age-Group ($F_{1,789}$ =

7.86, $p$ = .005, $\eta_p^2$ = .010) and User-Group*Age-Group ($F_{1,789}$ = 14.53, $p$ < .001, $\eta_p^2$ = .018). Post hoc ANCOVAs conducted separately for adults and adolescents for scores before and after lockdown suggested that adult users scored lower than adult controls both before ($F_{1,419}$ = 3.84, $p$ = .051, $\eta_p^2$ = .009) and after ($F_{1,419}$ = 13.05, $p$ < .001, $\eta_p^2$ = .030) lockdown, whereas adolescent users scored higher than adolescent controls before lockdown ($F_{1,365}$ = 4.71, $p$ = .03, $\eta_p^2$ = .013) but not after lockdown ($F_{1,365}$ = .11, $p$ = .74).

[FIGURE 1 APPROXIMATELY HERE]

**Figure 1**. Means and standard errors for the Apathy Evaluation Scale and Snaith-Hamilton Pleasure Scale by group before and after lockdown. Higher scores indicate higher levels of apathy and anhedonia, respectively.

Both main AES and SHAPS models were rerun excluding those subjects who changed user status from before to after lockdown, according to the cut-off at four days per month for users. Excluding these subjects did not have a marked impact on results (tables S5 and S6). There were no significant correlations between days/month of cannabis use and AES and SHAPS scores either before or after lockdown (Table S2), indicating that frequency of use was not related to apathy or anhedonia.

*3.3 Follow-up Analyses for SDS Scores*

Bivariate correlations revealed significant positive associations between SDS and AES scores before ($r$ = .21, $p$ < .001) and after ($r$ = .26, $p$ < .001) lockdown, and between SDS and SHAPS scores before ($r$ = .13, $p$ = .006) and after ($r$ = .22, $p$ < .001) lockdown in 452 cannabis users with a valid SDS score (see Table S2). In order to investigate this relationship more closely, we performed exploratory repeated-measures ANCOVAs identical to the main analyses, but with Dependence (yes/no) as determined by the SDS instead of User-Group as the main predictor. FDR correction with a priori $q < .10$ was used (Genovese et al., 2002). Four cannabis users were missing SDS scores, and were therefore not included in the exploratory analyses. Full results of these models are presented in Supplemental Table S7. Both models showed a significant between-subjects effect of Dependence ($F_{1,443} = 17.05$, $p < .001$, $\eta_p^2 = .037$ for AES, $F_{1,443} = 13.07$, $p < .001$, $\eta_p^2 = .029$ for SHAPS), indicating that dependent cannabis users had higher levels of apathy and anhedonia overall. Additionally, there was a significant Time*Dependence interaction effect in both models ($F_{1,443} = 4.47$, $p = .04$, $\eta_p^2 = .010$ for AES, $F_{1,443} = 4.25$, $p = .04$, $\eta_p^2 = .010$ for SHAPS), suggesting a greater increase in levels of apathy and anhedonia from before to after lockdown in dependent cannabis users. There were no significant interaction effects of Dependence*Age ($p = .36$ for AES, $p = .15$ for SHAPS) or Time*Dependence*Age ($p = .59$ for AES, $p = .96$ for SHAPS). The main effects of Age, and the Time*Age interaction effects, did not change markedly relative to the main models. All p-values below .05 survived the FDR correction. Figure 2 shows mean scores on the AES and SHAPS before and after lockdown by dependence and age.

[FIGURE 2 APPROXIMATELY HERE]

**Figure 2**. Means and standard errors for the Apathy Evaluation Scale and Snaith-Hamilton Pleasure Scale by level of dependence within cannabis users before and after lockdown. Higher scores indicate higher levels of apathy and anhedonia, respectively.

## 4. Discussion

*4.1 Apathy and Anhedonia in Adult and Adolescent Cannabis Users and Controls*

Adolescent cannabis users had the highest levels of apathy and anhedonia overall, but the difference between adolescent cannabis users and controls was only statistically significant for anhedonia before lockdown. Adolescent users scored on average 2.16 points higher than adolescent controls on the SHAPS, representing a small effect size ($\eta_p^2$ = .013). Conversely, adult cannabis users had significantly lower levels of both apathy and anhedonia compared to adult controls at both timepoints. The mean difference in scores between adult users and controls was marginal before lockdown for both the AES and SHAPS (<1 point, see Figure 1), corresponding to small effect sizes ($\eta_p^2$ = .009). The differences between adult users and controls after lockdown were substantially larger ($\eta_p^2$ = .048 for AES, $\eta_p^2$ = .030 for SHAPS), and are likely attributable to the larger increase in apathy and anhedonia levels among adult controls compared to adult users.

As adults used cannabis more frequently and were more dependent on average than adolescents, the difference between adult and adolescent user groups is unlikely to be due to differences in cannabis use. Rather, adolescent cannabis users might be more vulnerable to apathy compared to adult cannabis users, and to anhedonia compared to adult users and adolescent controls. There are several potential explanations for this enhanced vulnerability in adolescents. Adolescence is an important period of prolonged neuromaturation during which external factors, such as cannabis use, may be especially powerful in influencing brain and cognitive development (Bossong and Niesink, 2010; Lubman et al., 2015). Prolonged cannabis exposure during adolescence could contribute to blunted reward processing in the brain, increasing the susceptibility to anhedonia (Volkow et al., 2017). However, one large longitudinal study by Leventhal et al. (2017) found that cannabis use did not predict future anhedonia in adolescence, but rather anhedonia predicted future cannabis use. These results suggest that anhedonia might be a contributing factor to cannabis use in adolescence, or might co-occur with other factors which predict cannabis use.

Importantly, while adolescent cannabis users had higher levels of both apathy and anhedonia than the other groups, most differences between adolescent users and controls were not statistically significant after controlling for covariates. The effect of the depression covariate was large in both models (see tables S5 and S6), with a smaller, though statistically significant effect of anxiety. Current depression and anxiety were endorsed more frequently by adolescent users than by any other group. Thus, depression and anxiety might be more important than cannabis use in predicting blunted reward and motivation during adolescence.

*4.2 Increases in Apathy and Anhedonia During the COVID-19 Lockdown*

Our results showed that levels of both apathy and anhedonia increased during lockdown, as predicted by our first hypothesis. The increase in apathy was particularly large, corresponding to a medium-to-large effect size ($\eta_p^2$ = .096), and was significantly higher for adolescents compared to adults. Contrary to our second hypothesis, levels of apathy and anhedonia rose less for cannabis users than controls during the COVID-19 lockdown.

The adolescents in our sample reported COVID-19-related interruptions to their work or studies more frequently than adults, and a more negative mental health impact of COVID-19, on average (see Table 1). Thus, adolescents may have faced more severe disruptions to their life and mental health as a result of the pandemic, which could explain the greater increase in apathy in this age group. The unexpected smaller increase in scores among cannabis users compared to controls could be due to the fact that while lockdown has kept people from engaging in activities they normally find enjoyable, it has not prevented users from using cannabis. Indeed, cannabis users appear to have increased their use during the pandemic, partly in order to counteract the boredom that comes with being under strict social distancing regulations (Winstock et al., 2020). This was also the case in the present sample. Thus, for users, cannabis may have been an easily accessible coping strategy to deal with the monotony of lockdown. Additionally, cannabis users were less likely to report compliance with stay-at-home recommendations (see Table 1), which could have contributed to this result.

#### *4.3 Apathy and Anhedonia in Dependent and Non-dependent Cannabis Users*

Among cannabis users, those who were categorised as dependent on the SDS had significantly higher levels of apathy ($\eta_p^2$ = .037) and anhedonia ($\eta_p^2$ = .029) than those who were not dependent, corresponding to small-to-medium effect sizes. Levels of apathy and anhedonia also increased more for dependent compared to non-dependent users during the COVID-19 lockdown.

There were no significant correlations between days per month of cannabis use and AES or SHAPS scores within the user group (see Table S2). Higher levels in dependent users cannot, therefore, be attributed to higher use frequency. Instead, our results suggest that cannabis dependence is a more important predictor of apathy and anhedonia than cannabis use per se. Cannabis users who go on to develop dependence may have worse mental health compared to those who do not develop dependence (Marel et al., 2019), as well as potentially increased risk of depressive symptoms with chronic stress exposure (Sidhpura and Parsons, 2011; Volkow et al., 2017). In the current sample, dependent users did indeed report mental health problems more frequently than did non-dependent users, though current anxiety and depression were controlled in all analyses. Cannabis dependence might thus be a marker of vulnerability to mental ill-health, including apathy and anhedonia, which could also contribute to lower resilience to prolonged stressors like the COVID-19 pandemic. Additionally, dysregulated reward processing features centrally in theories of substance use disorder (e.g. Blum et al., 2000; Berridge and Robinson, 2016; Koob and Volkow, 2016). From this perspective, apathy and anhedonia might be considered characteristic of cannabis dependence, providing an alternative explanation of the present findings.

It is worth making a final remark on the relative effects of age and dependence on apathy and anhedonia. While we did not conduct a formal analysis comparing controls with dependent and non-dependent users, the results of the exploratory analyses suggest that the significant between-group differences in the main analyses were chiefly driven by adolescent dependent users and adult non-dependent users. Both adolescent age and cannabis dependence increased the vulnerability of cannabis users to apathy and anhedonia, and their effects appeared to be additive.

*4.4 Limitations*

The current study has several strengths including the large, well-balanced sample and adjustment with relevant confounders. An important limitation is the retrospective, rather than prospective assessment of pre-pandemic cannabis use and apathy and anhedonia. However, given the abrupt changes in circumstances of the pandemic lockdown, it was hard to avoid this limitation. Another limitation concerns group characteristics. Firstly, the adult age group included younger adults, which means the current findings may not generalise to older adults (>30 years). Secondly, although we controlled for several important covariates, we did not assess possible confounding by education, income, or employment status.

*4.5 Conclusion*

To our knowledge, this is the first study to look at the impact of the COVID-19 pandemic lockdown on a mental health outcome in cannabis users compared to controls. Several conclusions can be drawn. Firstly, our results suggest that adolescent cannabis users have higher levels of anhedonia compared to adolescent controls, and higher levels of apathy and anhedonia compared to adult users. There was no evidence of higher apathy or anhedonia in adult cannabis users, compared to controls. Secondly, within users, cannabis dependence is associated with significantly higher levels of both apathy and anhedonia. These results suggest that individual differences within cannabis users may be more important than user status per se, in predicting apathy and anhedonia with cannabis use, and that adolescent dependent users might be particularly vulnerable. Future studies should investigate which factors make some cannabis users especially vulnerable to apathy and anhedonia, in addition to age and dependence.

Our results also suggest that the COVID-19 lockdown has had a significant negative impact on hedonic processing and motivation, and that adolescents and people with substance dependence may be more vulnerable. Future research should continue to track the mental health of both cannabis users and controls after lockdown measures have ended, so that existing and novel treatment and prevention strategies can be rapidly employed to mitigate harmful outcomes.

## Tables

| | Adolescent users (*n* = 200) | Adolescent controls (*n* = 172) | Adult users (*n* = 256) | Adult controls (*n* = 170) | *p* (users vs. controls) |
|---|---|---|---|---|---|
| **Gender** f/m/other, n | 104/93/3 | 87/80/5 | 126/121/9 | 83/78/9 | |
| **Age in years,** mean (SD) | 16.73 (0.44) | 16.65 (0.48) | 22.38 (3.10) | 22.51 (3.50) | |
| **Alcohol days/month, mean (SD), range** | | | | | |
| Before lockdown | 5.17 (5.72), 0-30 | 3.43 (4.11), 0-24 | 7.40 (6.81), 0-30 | 6.71 (6.74), 0-30 | .002 |
| After lockdown | 6.60 (7.57), 0-30 | 4.13 (5.42), 0-25 | 8.20 (8.30), 0-30 | 6.64 (8.16), 0-30 | <.001 |
| **Cigarettes days/month, mean (SD), range** | | | | | |
| Before lockdown | 13.98 (12.92), 0-30 | 1.48 (5.59), 0-30 | 12.10 (13.12), 0-30 | 2.82 (7.75), 0-30 | <.001 |
| After lockdown | 12.35 (12.53), 0-30 | 1.13 (4.69), 0-30 | 9.72 (12.18), 0-30 | 1.91 (6.74), 0-30 | <.001 |
| **Illicit drugs, n (%)** | | | | | |
| Before lockdown | 103 (51.50) | 6 (3.49) | 102 (39.84) | 19 (11.18) | <.001 |
| After lockdown | 60 (30.00) | 3 (1.74) | 73 (28.52) | 15 (8.82) | <.001 |
| **Depression, n (%)** | | | | | |
| Yes, currently | 81 (41) | 41 (24) | 95 (37) | 36 (21) | <.001 |
| Yes, in the past | 56 (28) | 42 (24) | 90 (35) | 65 (38) | |
| No/never | 63 (32) | 89 (52) | 71 (28) | 69 (41) | |
| **Anxiety, n (%)** | | | | | |
| Yes, currently | 109 (54) | 66 (38) | 135 (53) | 65 (38) | <.001 |
| Yes, in the past | 35 (18) | 25 (15) | 55 (21) | 42 (25) | |
| No/never | 56 (28) | 81 (47) | 66 (26) | 63 (37) | |
| **COVID-19 impact on employment/study status, n (%)** | | | | | |
| 1 No change | 22 (11.00) | 10 (5.81) | 41 (16.02) | 17 (10.00) | |
| 2 Working from home | 45 (22.50) | 44 (25.58) | 67 (26.17) | 71 (41.76) | .37 |
| 3 Part/complete furlough, or disrupted/ postponed studies | 102 (51.00) | 102 (59.30) | 113 (44.14) | 66 (38.82) | |
| 4 Lost job or terminated studies | 31 (15.50) | 16 (9.30) | 35 (13.68) | 16 (9.41) | |
| COVID-19 impact on mental health, mean (SD) | -1.74 (2.55) | -1.59 (2.34) | -1.61 (2.34) | -1.29 (2.37) | .20 |
| **Had COVID-19, n (%)** | | | | | |
| Yes | 3 (1.50) | 2 (1.16) | 5 (1.95%) | 2 (1.18) | .14 |
| Suspected | 43 (21.50) | 36 (20.93) | 54 (21.09) | 24 (14.12) | |

| | | | | | |
|---|---|---|---|---|---|
| No | 154 (77.00) | 134 (77.91) | 197 (76.95) | 144 (84.71) | |
| Lost a loved one to COVID-19, n (%) | 15 (7.50) | 13 (7.56) | 11 (4.30) | 7 (4.12) | .93 |
| Currently following stay-at-home recommendations, n (%) | 47 (23.5) | 99 (57.6) | 147 (57.4) | 117 (68.86) | <.001 |

**Table 1.** Sample characteristics

*Abbreviations.* f – female, m – male

Note. Other drug use classified as yes if the participant had used any of the following drugs at least once per month over the past three months: 3,4-Methylenedioxymethamphetamine (MDMA), cocaine, nitrous oxide (laughing gas), ketamine, psilocybin/magic mushrooms, lysergic acid diethylamide (LSD), methamphetamine, heroin. User-Group comparisons were performed with independent samples *t*-tests for scaled variables, and with Pearson chi-square tests for binarized count variables (current depression yes/no, current anxiety yes/no, COVID-19 employment/study categories 1 and 2 vs. 3 and 4, had or suspected COVID-19 yes/no).

| | **Adolescent users** (*n* = 200) | **Adolescent controls** (*n* = 172) | **Adult users** (*n* = 256) | **Adult controls** (*n* = 170) | ***p*** (adults vs. adolescents) |
|---|---|---|---|---|---|
| Days/month use before lockdown, mean (SD), range | 13.92 (8.65), 4-30 | 0.23 (0.63), 0-3 | 18.98 (9.59), 4-30 | 0.44 (0.85), 0-3 | <.001 (users) .01 (controls) |
| Days/month use after lockdown, mean (SD), range | 16.90 (10.90), 0-30 | 0.65 (2.46), 0-20 | 20.66 (10.44), 0-30 | 1.24 (3.92), 0-25 | <.001 (users) .09 (controls) |
| Ever use, n (%) | NA | 65 (37.79) | NA | 105 (61.76) | <.001 |
| Ever regular use, n (%) | NA | 7 (4.07) | NA | 41 (24.12) | <.001 |
| Age of first use, mean (SD) | 14.18 (1.18) | 14.86 (1.00) *n* = 65 | 16.20 (2.04) | 16.75 (2.42) *n* = 105 | <.001 (users) <.001 (controls) |
| Age of regular use, mean (SD) | 15.84 (0.91) *n* = 199 | 15.71 (0.95) *n* = 7 | 19.11 (2.42) *n* = 253 | 17.80 (2.79) *n* = 41 | <.001 (users) .001 (controls) |
| SDS, mean (SD) | 3.02 (2.96) *n* = 199 | 2.17 (1.33) *n* = 6 | 3.53 (3.32) *n* = 253 | 2.18 (1.94) *n* = 17 | .09 (users) .99 (controls) |

**Table 2.** Cannabis use variables

*Abbreviations.* NA – not applicable, SDS – Severity of Dependence Scale

Note. The SDS is reported for controls who used cannabis ≥4 days per month after lockdown. Age-Group comparisons were made within users and controls, with independent samples *t*-tests for scaled variables, and Pearson chi-square tests for count variables.

### Funding

This work was supported by Eton College (grant number RNAG403). MS is funded by an Aker Scholarship from the Aker Foundation. BJS receives funding from the Wallitt Foundation, Eton College, and a Wellcome Trust Collaborative Award 200181/Z//15/Z, CL is funded by a Wellcome Trust Collaborative Award 200181/Z//15/Z, and their research is conducted within the NIHR Cambridge Biomedical Research Centre (Mental Health Theme and Neurodegeneration Theme) and the NIHR Brain Injury MedTech and in vitro diagnostics Co-operative (MIC), Cambridge.

### Acknowledgements

We thank Natalie Valle Guzman for her help with setting up the study, and Katherine Petrilli for her help with data collection.

### Statement of Interest

BJS consults for Cambridge Cognition and Greenfield BioVentures. VV consults for Boehringer Ingelheim. Remaining authors have no conflicts of interest to disclose.

Figure 1

Accepted Manus

Figure 2

## Supplementary Material

pyab033_suppl_Supplementary_Materials

