# Supplementary material for: Apathy and Anhedonia in Adult and Adolescent Cannabis Users and Controls Before and During the COVID-19 Pandemic Lockdown
Source: Int J Neuropsychopharmacol. 2021 Jun 2;24(11):859–66. doi: 10.1093/ijnp/pyab033 (PMC8244616; doi:10.1093/ijnp/pyab033)
Supplement: pyab033_suppl_Supplemental_Materials [file pyab033_suppl_supplemental_materials.docx]

# **Supplemental materials**


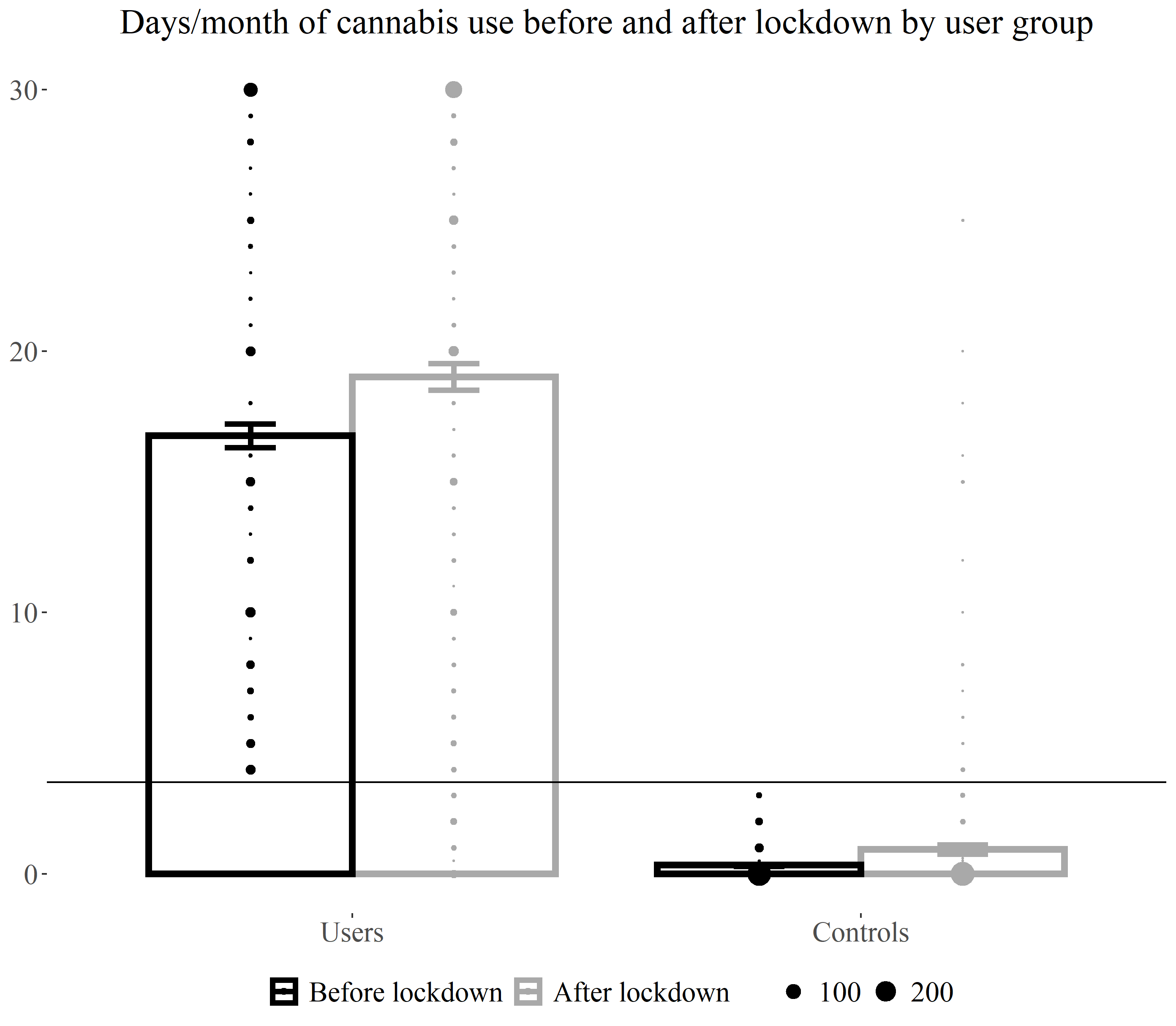


**Figure S1.** Means and standard errors of days per month of cannabis use by group before and after lockdown. Dots represent participants, with the diameter indicating the frequency of participants reporting the given value. The horizonal line at y = 3.5 shows the cut-off between users and controls.

**Table S2.** Bivariate Pearson correlations

| **Factor** | AES  before lockdown | AES  after lockdown | SHAPS  before lockdown | SHAPS  after lockdown |
| --- | --- | --- | --- | --- |
| *Full sample*  *n = 798* |  |  |  |  |
| Age | -.108** | -.172*** | -.132*** | -.107** |
| DPM alcohol use | -.020 | .004 | -.051 | -.029 |
| DPM cigarette use | .089* | .093** | .080* | .072* |
| Illicit drug use | -.004 | -.020 | -.020 | -.035 |
| Depression | .338*** | .311*** | .308*** | .303*** |
| Anxiety | 234*** | .230*** | .223*** | 222*** |
| *Cannabis users*  *n = 456* |  |  |  |  |
| Cannabis DPM before lockdown | .072 | .012 | .009 | .033 |
| Cannabis DPM after lockdown | .054 | -.015 | .036 | -.039 |
| Age of first use | -.039 | -.099* | -.086 | -.038 |
| Age of regular use, *n* = 452 | -.120* | -.188*** | -.171*** | -.156** |
| SDS,  *n* = 452 | .210*** | .258*** | .128** | .218*** |

**p* < .05

***p* < .01

****p* < .001

*Abbreviations.* AES – Apathy Evaluation scale, DPM – days per month, SHAPS – Snaith-Hamilton Pleasure Scale, SDS – Severity of Dependence Scale


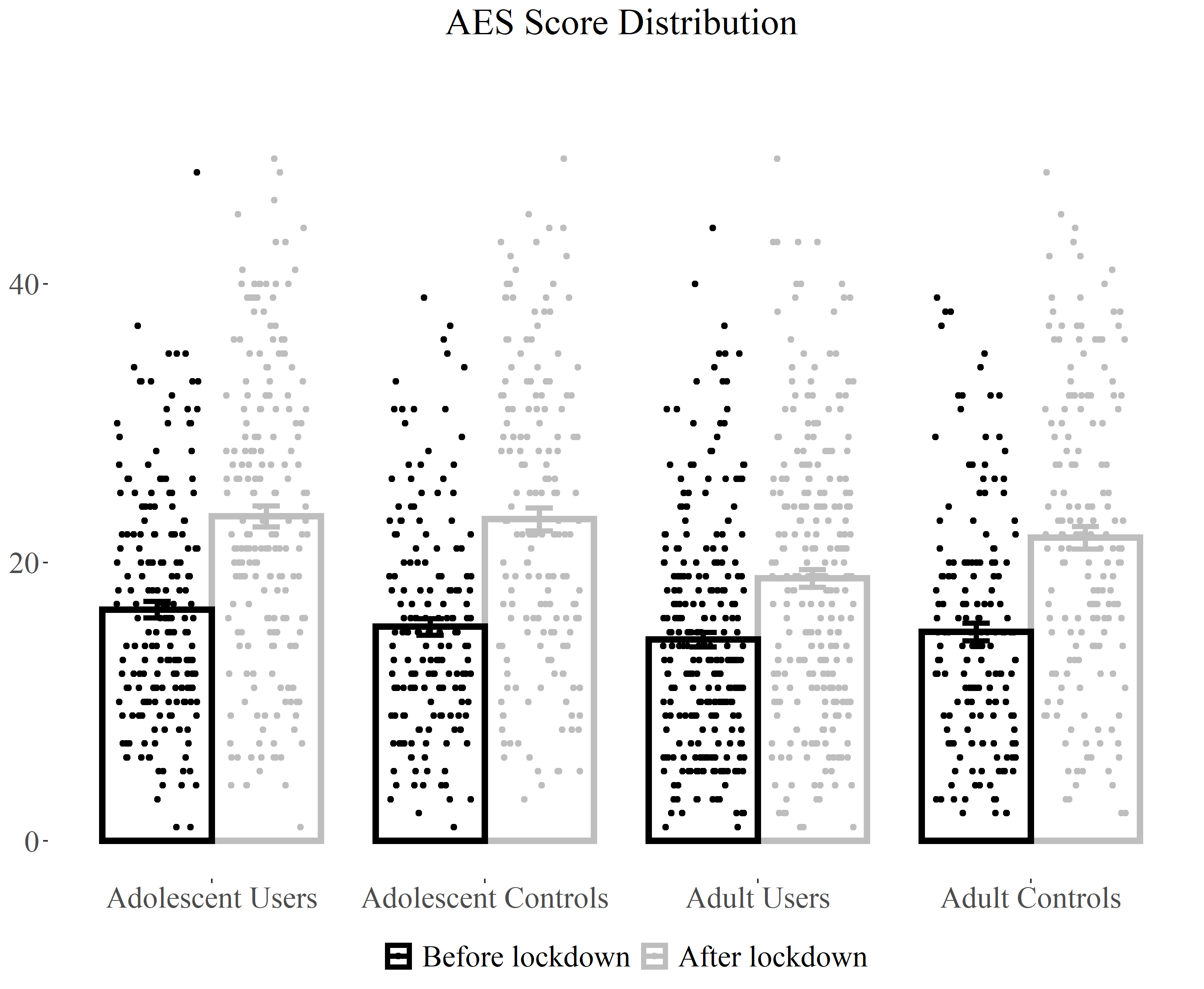


**Figure S3.** Means and standard errors for the Apathy Evaluation Scale by group before and after lockdown, with individual scores overlayed.


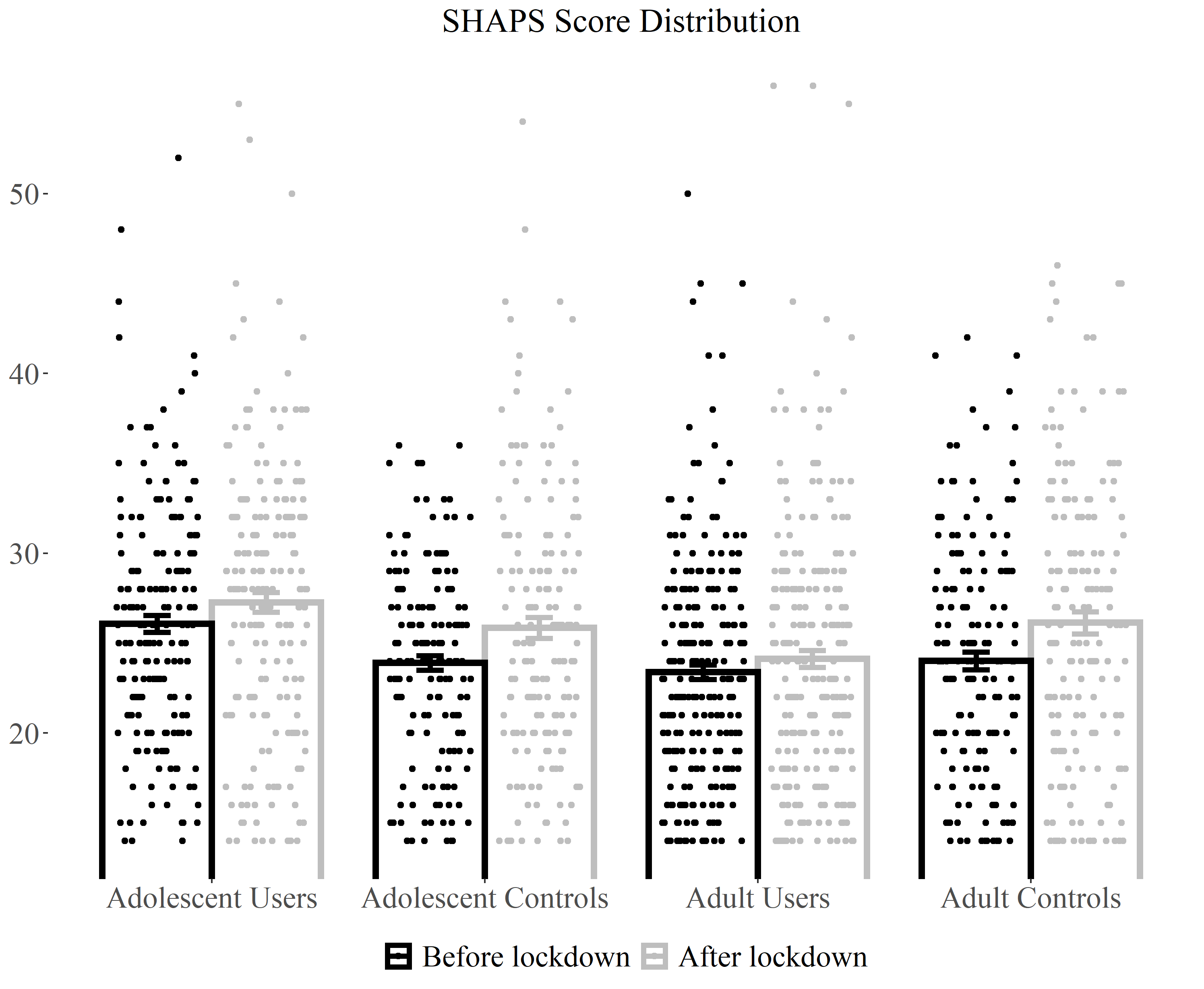


**Figure S4.** Means and standard errors for the Snaith-Hamilton Pleasure Scale by group before and after lockdown, with individual scores overlayed.

**Table S5.** AES complete results for full and partial sample

|  | Full sample, *n* = 798 | | | |  | Partial sample, *n* = 708 | | | |
| --- | --- | --- | --- | --- | --- | --- | --- | --- | --- |
|  | **F** | **df** | **p** | **η_p_^2^** |  | **F** | **df** | **p** | **η_p_^2^** |
| Time | 83.44 | 1, 789 | <.001 | .096 |  | 67.20 | 1, 699 | <.001 | .088 |
| User-Group | 11.89 | 1, 789 | .001 | .015 |  | 14.77 | 1, 699 | <.001 | .021 |
| Age-Group | 11.27 | 1, 789 | .001 | .014 |  | 11.45 | 1, 699 | .001 | .016 |
| Alcohol | 0.14 | 1, 789 | .71 | <.001 |  | 0.08 | 1, 699 | .77 | <.001 |
| Cigarettes | 3.83 | 1, 789 | .051 | .005 |  | 5.03 | 1, 699 | .03 | .007 |
| Illicit drugs | 0.59 | 1, 789 | .44 | .001 |  | 0.27 | 1, 699 | .60 | <.001 |
| Depression | 72.31 | 1, 789 | <.001 | .084 |  | 66.71 | 1, 699 | <.001 | .087 |
| Anxiety | 11.88 | 1, 789 | .001 | .015 |  | 12.71 | 1, 699 | <.001 | .018 |
| Time*User-Group | 11.22 | 1, 789 | .001 | .014 |  | 17.89 | 1, 699 | <.001 | .025 |
| Time*Age-Group | 6.19 | 1, 789 | .01 | .008 |  | 4.12 | 1, 699 | .04 | .006 |
| Time*Alcohol | 1.37 | 1,789 | .24 | .002 |  | 2.35 | 1, 699 | .13 | .003 |
| Time*Cigarettes | 2.22 | 1, 789 | .14 | .003 |  | 3.52 | 1, 699 | .07 | .005 |
| Time*Illicit drugs | 0.004 | 1, 789 | .95 | <.001 |  | 0.72 | 1, 699 | .40 | .001 |
| Time*Depression | 1.17 | 1, 789 | .28 | .001 |  | 1.02 | 1, 699 | .31 | .001 |
| Time*Anxiety | 1.38 | 1, 789 | .24 | .002 |  | 1.22 | 1, 699 | .27 | .002 |
| User-Group*Age-Group | 4.19 | 1, 789 | .04 | .005 |  | 4.37 | 1, 699 | .04 | .006 |
| Time*User-Group*Age-Group | 0.70 | 1, 789 | .40 | .001 |  | <0.001 | 1, 699 | .998 | <.001 |

Note. The partial sample excluded controls using cannabis more than three days per month after lockdown (*n* = 25) and users using cannabis less than four days per month after lockdown (*n* = 65).

**Table S6.** SHAPS complete results for full and partial sample

|  | Full sample, *n* = 798 | | | |  | Partial sample, *n* = 708 | | | |
| --- | --- | --- | --- | --- | --- | --- | --- | --- | --- |
|  | **F** | **df** | **p** | **η_p_^2^** |  | **F** | **df** | **p** | **η_p_^2^** |
| Time | 3.54 | 1, 789 | .06 | .004 |  | 0.55 | 1, 699 | .46 | .001 |
| User-Group | 1.48 | 1, 789 | .23 | .002 |  | 3.80 | 1, 699 | .052 | .005 |
| Age-Group | 7.86 | 1, 789 | .01 | .010 |  | 10.80 | 1, 699 | .001 | .015 |
| Alcohol | 0.67 | 1, 789 | .41 | .001 |  | 0.03 | 1, 699 | .86 | <.001 |
| Cigarettes | 0.84 | 1, 789 | .36 | .001 |  | 1.76 | 1, 699 | .19 | .003 |
| Illicit drugs | 2.50 | 1, 789 | .11 | .003 |  | 2.23 | 1, 699 | .14 | .003 |
| Depression | 63.32 | 1, 789 | <.001 | .074 |  | 61.16 | 1, 699 | <.001 | .020 |
| Anxiety | 11.09 | 1, 789 | .001 | .014 |  | 14.31 | 1, 699 | <.001 | .026 |
| Time*User-Group | 6.49 | 1, 789 | .01 | .008 |  | 16.17 | 1, 699 | <.001 | .023 |
| Time*Age-Group | 0.07 | 1, 789 | .80 | <.001 |  | 0.13 | 1, 699 | .72 | <.001 |
| Time*Alcohol | 0.42 | 1,789 | .52 | .001 |  | 0.76 | 1, 699 | .38 | .001 |
| Time*Cigarettes | 0.64 | 1, 789 | .42 | .001 |  | 2.49 | 1, 699 | .12 | .004 |
| Time*Illicit drugs | 0.04 | 1, 789 | .84 | <.001 |  | 1.26 | 1, 699 | .26 | .002 |
| Time*Depression | 1.92 | 1, 789 | .17 | .002 |  | 1.86 | 1, 699 | .17 | .003 |
| Time*Anxiety | 0.55 | 1, 789 | .46 | .001 |  | 0.26 | 1, 699 | .61 | <.001 |
| User-Group*Age-Group | 14.53 | 1, 789 | <.001 | .018 |  | 19.03 | 1, 699 | <.001 | .026 |
| Time*User-Group*Age-Group | 0.28 | 1, 789 | .60 | <.001 |  | 0.11 | 1, 699 | .74 | <.001 |

Note. The partial sample excluded controls using cannabis more than three days per month after lockdown (*n* = 25) and users using cannabis less than four days per month after lockdown (*n* = 65).

**Table S7.** Complete results of exploratory analyses of dependence in *n* = 452 cannabis users

|  | AES | | | |  | SHAPS | | | |
| --- | --- | --- | --- | --- | --- | --- | --- | --- | --- |
|  | **F** | **df** | **p** | **η_p_^2^** |  | **F** | **df** | **p** | **η_p_^2^** |
| Time | 22.66 | 1, 443 | <.001 | .049 |  | 1.03 | 1, 443 | .31 | .002 |
| Dependence | 17.05 | 1, 443 | <.001 | .037 |  | 13.07 | 1, 443 | <.001 | .029 |
| Age-Group | 23.99 | 1, 443 | <.001 | .051 |  | 31.23 | 1, 443 | <.001 | .066 |
| Alcohol | 0.13 | 1, 443 | .72 | <.001 |  | 0.19 | 1, 443 | .66 | <.001 |
| Cigarettes | 0.52 | 1, 443 | .47 | .001 |  | 0.004 | 1, 443 | .95 | <.001 |
| Illicit drugs | 3.59 | 1, 443 | .06 | .008 |  | 6.18 | 1, 443 | .01 | .014 |
| Depression | 39.24 | 1, 443 | <.001 | .081 |  | 33.91 | 1, 443 | <.001 | .071 |
| Anxiety | 1.61 | 1, 443 | .21 | .004 |  | 2.14 | 1, 443 | .15 | .005 |
| Time*Dependence | 4.47 | 1, 443 | .04 | .010 |  | 4.25 | 1, 443 | .04 | .010 |
| Time*Age-Group | 8.23 | 1, 443 | .004 | .018 |  | 1.04 | 1, 443 | .31 | .002 |
| Time*Alcohol | 1.95 | 1, 443 | .16 | .004 |  | 0.74 | 1, 443 | .39 | .002 |
| Time*Cigarettes | 0.57 | 1, 443 | .45 | .001 |  | 0.10 | 1, 443 | .76 | <.001 |
| Time*Illicit drugs | 0.06 | 1, 443 | .81 | <.001 |  | 0.08 | 1, 443 | .78 | <.001 |
| Time*Depression | 2.03 | 1, 443 | .16 | .005 |  | 1.78 | 1, 443 | .18 | .004 |
| Time*Anxiety | 0.01 | 1, 443 | .93 | <.001 |  | 0.54 | 1, 443 | .46 | .001 |
| Dependence*Age-Group | 0.85 | 1, 443 | .36 | .002 |  | 2.12 | 1, 443 | .15 | .005 |
| Time*Dependence*Age-Group | 0.29 | 1, 443 | .59 | .001 |  | 0.002 | 1, 443 | .96 | <.001 |
