# Supplementary material for: Apathy and anhedonia in adult and adolescent cannabis users and controls before and during the COVID-19 pandemic lockdown
Source: Int J Neuropsychopharmacol. 2021 Jun 2:pyab033. doi: 10.1093/ijnp/pyab033 (PMC8244616; doi:10.1093/ijnp/pyab033)
Supplement: Supplementary file 1 [file pyab033_suppl_supplementary_materials.docx]

# **Supplemental materials**

**Figure S****1****.** Means and standard errors of days per month of cannabis use by group before and after lockdown. Dots represent participants, with the diameter indicating the frequency of participants reporting the given value. The horizonal line at y = 3.5 shows the cut-off between users and controls. 

**Table S****2****.** Bivariate Pearson correlations 

| **Factor** | AES before lockdown | AES after lockdown | SHAPS before lockdown | SHAPS after lockdown |
| --- | --- | --- | --- | --- |
| *Full sample* *n = 798* |  |  |  |  |
| Age | -.108** | -.172*** | -.132*** | -.107** |
| DPM alcohol use | -.020 | .004 | -.051 | -.029 |
| DPM cigarette use | .089* | .093** | .080* | .072* |
| Illicit drug use | -.004 | -.020 | -.020 | -.035 |
| Depression | .338*** | .311*** | .308*** | .303*** |
| Anxiety | 234*** | .230*** | .223*** | 222*** |
| *Cannabis users* *n = 456* |  |  |  |  |
| Cannabis DPM before lockdown | .072 | .012 | .009 | .033 |
| Cannabis DPM after lockdown | .054 | -.015 | .036 | -.039 |
| Age of first use | -.039 | -.099* | -.086 | -.038 |
| Age of regular use, *n* = 452 | -.120* | -.188*** | -.171*** | -.156** |
| SDS,  *n* = 452 | .210*** | .258*** | .128** | .218*** |

**p* < .05

***p* < .01

****p* < .001

*Abbreviations.* AES – Apathy Evaluation scale, DPM – days per month, SHAPS – Snaith-Hamilton Pleasure Scale, SDS – Severity of Dependence Scale

**Figure S****3****.** Means and standard errors for the Apathy Evaluation Scale by group before and after lockdown, with individual scores overlayed.

**Figure S****4****.** Means and standard errors for the Snaith-Hamilton Pleasure Scale by group before and after lockdown, with individual scores overlayed.

**Table S****5****.** AES complete results for full and partial sample

|  | Full sample, *n* = 798 |  |  |  |  | Partial sample, *n* = 708 |  |  |  |
| --- | --- | --- | --- | --- | --- | --- | --- | --- | --- |
|  | **F** | **df** | **p** | **η**_**p**_^**2**^ |  | **F** | **df** | **p** | **η**_**p**_^**2**^ |
| Time | 83.44 | 1, 789 | <.001 | .096 |  | 67.20 | 1, 699 | <.001 | .088 |
| User-Group | 11.89 | 1, 789 | .001 | .015 |  | 14.77 | 1, 699 | <.001 | .021 |
| Age-Group | 11.27 | 1, 789 | .001 | .014 |  | 11.45 | 1, 699 | .001 | .016 |
| Alcohol | .14 | 1, 789 | .71 | <.001 |  | .08 | 1, 699 | .77 | <.001 |
| Cigarettes | 3.83 | 1, 789 | .051 | .005 |  | 5.03 | 1, 699 | .03 | .007 |
| Illicit drugs | .59 | 1, 789 | .44 | .001 |  | .27 | 1, 699 | .60 | <.001 |
| Depression | 72.31 | 1, 789 | <.001 | .084 |  | 66.71 | 1, 699 | <.001 | .087 |
| Anxiety | 11.88 | 1, 789 | .001 | .015 |  | 12.71 | 1, 699 | <.001 | .018 |
| Time*User-Group | 11.22 | 1, 789 | .001 | .014 |  | 17.89 | 1, 699 | <.001 | .025 |
| Time*Age-Group | 6.19 | 1, 789 | .01 | .008 |  | 4.12 | 1, 699 | .04 | .006 |
| Time*Alcohol | 1.37 | 1,789 | .24 | .002 |  | 2.35 | 1, 699 | .13 | .003 |
| Time*Cigarettes | 2.22 | 1, 789 | .14 | .003 |  | 3.52 | 1, 699 | .07 | .005 |
| Time*Illicit drugs | .004 | 1, 789 | .95 | <.001 |  | .72 | 1, 699 | .40 | .001 |
| Time*Depression | 1.17 | 1, 789 | .28 | .001 |  | 1.02 | 1, 699 | .31 | .001 |
| Time*Anxiety | 1.38 | 1, 789 | .24 | .002 |  | 1.22 | 1, 699 | .27 | .002 |
| User-Group*Age-Group | 4.19 | 1, 789 | .04 | .005 |  | 4.37 | 1, 699 | .04 | .006 |
| Time*User-Group*Age-Group | .70 | 1, 789 | .40 | .001 |  | <.001 | 1, 699 | .998 | <.001 |

Note. The partial sample excluded controls using cannabis more than three days per month after lockdown (*n* = 25) and users using cannabis less than four days per month after lockdown (*n* = 65).

**Table S****6****.** SHAPS complete results for full and partial sample

 

|  | Full sample, *n* = 798 |  |  |  |  | Partial sample, *n* = 708 |  |  |  |
| --- | --- | --- | --- | --- | --- | --- | --- | --- | --- |
|  | **F** | **df** | **p** | **η**_**p**_^**2**^ |  | **F** | **df** | **p** | **η**_**p**_^**2**^ |
| Time | 3.54 | 1, 789 | .06 | .004 |  | .55 | 1, 699 | .46 | .001 |
| User-Group | 1.48 | 1, 789 | .23 | .002 |  | 3.80 | 1, 699 | .052 | .005 |
| Age-Group | 7.86 | 1, 789 | .01 | .010 |  | 10.80 | 1, 699 | .001 | .015 |
| Alcohol | .67 | 1, 789 | .41 | .001 |  | .03 | 1, 699 | .86 | <.001 |
| Cigarettes | .84 | 1, 789 | .36 | .001 |  | 1.76 | 1, 699 | .19 | .003 |
| Illicit drugs | 2.50 | 1, 789 | .11 | .003 |  | 2.23 | 1, 699 | .14 | .003 |
| Depression | 63.32 | 1, 789 | <.001 | .074 |  | 61.16 | 1, 699 | <.001 | .020 |
| Anxiety | 11.09 | 1, 789 | .001 | .014 |  | 14.31 | 1, 699 | <.001 | .026 |
| Time*User-Group | 6.49 | 1, 789 | .01 | .008 |  | 16.17 | 1, 699 | <.001 | .023 |
| Time*Age-Group | .07 | 1, 789 | .80 | <.001 |  | .13 | 1, 699 | .72 | <.001 |
| Time*Alcohol | .42 | 1,789 | .52 | .001 |  | .76 | 1, 699 | .38 | .001 |
| Time*Cigarettes | .64 | 1, 789 | .42 | .001 |  | 2.49 | 1, 699 | .12 | .004 |
| Time*Illicit drugs | .04 | 1, 789 | .84 | <.001 |  | 1.26 | 1, 699 | .26 | .002 |
| Time*Depression | 1.92 | 1, 789 | .17 | .002 |  | 1.86 | 1, 699 | .17 | .003 |
| Time*Anxiety | .55 | 1, 789 | .46 | .001 |  | .26 | 1, 699 | .61 | <.001 |
| User-Group*Age-Group | 14.53 | 1, 789 | <.001 | .018 |  | 19.03 | 1, 699 | <.001 | .026 |
| Time*User-Group*Age-Group | .28 | 1, 789 | .60 | <.001 |  | .11 | 1, 699 | .74 | <.001 |

Note. The partial sample excluded controls using cannabis more than three days per month after lockdown (*n* = 25) and users using cannabis less than four days per month after lockdown (*n* = 65).

**Table S****7****.** Complete results of exploratory analyses of dependence within *n* = 452 cannabis users

|  | AES |  |  |  |  | SHAPS |  |  |  |
| --- | --- | --- | --- | --- | --- | --- | --- | --- | --- |
|  | **F** | **df** | **p** | **η**_**p**_^**2**^ |  | **F** | **df** | **p** | **η**_**p**_^**2**^ |
| Time | 22.66 | 1, 443 | <.001 | .049 |  | 1.03 | 1, 443 | .31 | .002 |
| Dependence | 17.05 | 1, 443 | <.001 | .037 |  | 13.07 | 1, 443 | <.001 | .029 |
| Age-Group | 23.99 | 1, 443 | <.001 | .051 |  | 31.23 | 1, 443 | <.001 | .066 |
| Alcohol | .13 | 1, 443 | .72 | <.001 |  | .19 | 1, 443 | .66 | <.001 |
| Cigarettes | .52 | 1, 443 | .47 | .001 |  | .004 | 1, 443 | .95 | <.001 |
| Illicit drugs | 3.59 | 1, 443 | .06 | .008 |  | 6.18 | 1, 443 | .01 | .014 |
| Depression | 39.24 | 1, 443 | <.001 | .081 |  | 33.91 | 1, 443 | <.001 | .071 |
| Anxiety | 1.61 | 1, 443 | .21 | .004 |  | 2.14 | 1, 443 | .15 | .005 |
| Time*Dependence | 4.47 | 1, 443 | .04 | .010 |  | 4.25 | 1, 443 | .04 | .010 |
| Time*Age-Group | 8.23 | 1, 443 | .004 | .018 |  | 1.04 | 1, 443 | .31 | .002 |
| Time*Alcohol | 1.95 | 1, 443 | .16 | .004 |  | .74 | 1, 443 | .39 | .002 |
| Time*Cigarettes | .57 | 1, 443 | .45 | .001 |  | .10 | 1, 443 | .76 | <.001 |
| Time*Illicit drugs | .06 | 1, 443 | .81 | <.001 |  | .08 | 1, 443 | .78 | <.001 |
| Time*Depression | 2.03 | 1, 443 | .16 | .005 |  | 1.78 | 1, 443 | .18 | .004 |
| Time*Anxiety | .01 | 1, 443 | .93 | <.001 |  | .54 | 1, 443 | .46 | .001 |
| Dependence*Age-Group | .85 | 1, 443 | .36 | .002 |  | 2.12 | 1, 443 | .15 | .005 |
| Time*Dependence*Age-Group | .29 | 1, 443 | .59 | .001 |  | .002 | 1, 443 | .96 | <.001 |
